# Two commentaries on ‘Impact of tuberculosis preventive therapy on tuberculosis and mortality in HIV-infected children’

**DOI:** 10.1002/ebch.437

**Published:** 2009-12-16

**Authors:** Hoosen Coovadia, Ruth M Bland, Tony Walls

**Affiliations:** 1 Victor Daitz Professor of HIV/AIDS Research, University of KwaZulu-NatalDurban, South Africa; 2 Director HIV Management, Reproductive Health and HIV Research Unit, Durban Branch, University of the Witwatersrand; 3 Africa Centre for Health and Population Studies, University of kwaZulu-NatalSomkhele, KwaZulu-Natal, South Africa; 4 Division of Developmental Medicine, Medical Faculty, University of GlasgowUK; 5 University of New South Wales, Department of Immunology and Infectious Diseases, Sydney Children's HospitalSydney, Australia

## Commentary by Hoosen Coovadia and Ruth M. Bland

The concurrence of the two major epidemics of HIV and tuberculosis in many parts of the world has had a major impact on child health and survival^1^,^2^, reversing significant advances in promoting child health programmes in the southern African region. The HIV epidemic is pivotal in contributing to these losses, and tuberculosis is recognized as one of the most common opportunisitic infections^3^–^6^. In South Africa the plight of infants and children has worsened because of the impact of a number of colliding health epidemics, considered characteristic of poverty and wealth, on this age group (including maternal, newborn and child diseases; HIV/TB; non-communicable diseases; and violence and crime)^7^.

Considerable progress in paediatric HIV has been made since 1994 when antiretroviral drugs were first shown to reduce transmission of HIV from mothers to their infants^8^,^9^. Treatment of HIV-infected children has since been successfully introduced in many developing countries^10^,^11^. The current priorities in these countries are to increase coverage and improve the quality of health services. The increasing burden of tuberculosis in children *pari passu* with that of HIV, however, has not attracted the same degree of attention, despite the fact that co-infection with tuberculosis is a very serious disease. In comparison with HIV-uninfected children, tuberculosis in HIV-infected children is more readily acquired but more difficult to diagnose; has a higher incidence; disease is severe, with more rapid progression to death, higher mortality rates, lower cure rates and more frequent recurrences; and treatment is complicated by adverse drug interactions^2^.

The review by Gray, Zar and Cotton, is therefore particularly pertinent. The review describes the search for evidence of the benefit of INH prophylaxis in HIV-infected children. A stark reminder of the paucity of reliable data on this subject is that only one trial met their selection criteria.

The objectives of the study are well defined, and the search strategy was comprehensive, covering recognized web-based databases, manual searches for articles, and consultation with experts. They included studies of ‘HIV-infected children randomized to receive either tuberculosis prophylaxis or placebo, or an alternative TB preventive regimen’. One of the authors and an independent individual undertook the data extraction from the single study included in the review. The key question is whether the recommendations from the review are clinically useful and of public health importance. The authors conclude that ‘isoniazid prophylaxis has a substantial protective effect on TB incidence and death in HIV-infected children not on HAART’. The authors are rightly more circumspect in recommending ‘isoniazid prophylaxis in HIV-infected children has the potential to play a major public health role by reducing TB incidence and death’. The reason for their caution is not that there may be limitations to interpretation of the data from their single study, but because of untested issues including relevance of the findings to areas of low tuberculosis prevalence, absence of data on INH for children on HAART, and lack of clarity on the duration of INH prophylaxis.

The study has many strengths: it was well conducted; the design was appropriate; the diagnostic criteria, always difficult in children and more so when there is coinfection with HIV, are standard; the authors are among the leading figures in childhood tuberculosis; and the environment of their study has been extensively researched over many years. The statistical planning to determine sample size and the findings on efficacy, safety and tolerability are convincing.

What, if any, are the limitations? These are primarily because of the restricted applicability of the findings. There are different views on the number of studies required to initiate or change policy. Given the multiple variations in populations and their environments, it is prudent to expect divergent results from a range of settings. There were a number of publications from across continents on the use of Vitamin A prophylaxis to reduce child mortality and morbidity, which finally led to introduction at national level^12^. In contrast, the demonstration that antiretroviral drugs substantially reduced mother-to-child transmission in one study from the developed world changed practices in most countries^8^. However, the regimen used to achieve decreased transmission in developing countries differed markedly from the original protocol of the US/French study^13^,^14^. The authors of this review allude to the distinctive features at the study site, which should be considered before accepting any recommendation on INH prophylaxis for HIV-infected children. In summary these include: the extremely high tuberculosis incidence, one of the highest in the world; the relatively high socio-economic status of the population compared to the rest of South Africa; the superior health facilities compared to other provinces in the country; and background diseases such as alcoholism which are a dominant part of the profile of diseases.

Some minor points also require clarification, including: the effect of prior BCG vaccination; the proportion of children with ‘probable’ and ‘definite’ tuberculosis among those who became infected, and specifically whether the sensitivity of the definition of ‘probable’ cases affects the outcomes; the causes of mortality in the two study arms; and some discussion of the importance of determining whether INH prophylaxis is efficacious in HIV-exposed but uninfected infants, and when treatment in these infants should be started. This latter group of infants is increasingly relevant as Prevention of Mother-to-Child transmission programmes are implemented and vertical transmission is reduced. It would be useful to know if there are any published accounts of the study cohort 5 years after the publication of the paper, and pertinent to discuss the cost-benefits of rolling-out INH prophylaxis to all children, or whether it would be more effective to target certain groups of children, for example those living in households with tuberculosis.

Published results of other trials are awaited. A recent NIH funded study of INH prophylaxis in HIV-infected and HIV-exposed but uninfected infants, conducted in the Western Cape, Johannesburg and Durban, South Africa, failed to demonstrate any protection against incident tuberculosis in either of these two groups (reported in a plenary presentation by S. Madhi at the 1st International Workshop of Pediatrics, Cape Town, 17–18 July 2009). The authors of this review are cognizant of the need to restrict INH prophylaxis to those who cannot access antiretroviral treatment. The global effort to increase coverage will inevitably lead to wider access to these drugs, a decrease in tuberculosis incidence in those treated with HAART^15^, and probably discourage use of other prophylaxic drugs unless further work shows synergy.

## Declarations of Interest

None.

## Commentary by Tony Walls

Tuberculosis (TB) is a leading cause of HIV related deaths worldwide. In 2007 the WHO estimated that 1.37 million new cases of TB occurred among HIV-infected people with approximately 456,000 of them dying. The use of antiretroviral therapy in children has been found to reduce the probability of being diagnosed with TB by as much as 85%. Yet the majority of children with HIV who require antiretroviral therapy do not have access to it. Recent estimates suggest that only around 30% of people in sub-Saharan Africa who require antiretroviral therapy actually receive it. While there are ongoing improvements in access to HIV therapy in many regions, the proportion of children who receive appropriate treatment is often still well below the level in adults. There is therefore a real need for simple interventions to prevent TB disease in children with HIV infection who are at high risk of exposure to TB.

The use of isoniazid prophylaxis to prevent tuberculosis disease in patients with M. tuberculosis infection is well established in both adults and children. Following TB infection, children—especially those less than 2 years of age—are the most likely to develop disease. They are also more likely to develop severe disease such as miliary TB or TB meningitis. Hence, it is children who are most likely to benefit from any intervention that reduces the development of TB disease. This Cochrane review addresses the question of how effective isoniazid prophylaxis is for preventing TB in children with HIV infection who are at high-risk of TB exposure.

The major finding of the review is the lack of research on the use of isoniazid prophylaxis in HIV infected children. Only one study fulfilled the inclusion criteria for review, and this was a randomized controlled trial conducted by two of the review authors. They found significant benefits for children on isonaizid prophylaxis both in terms of all-cause mortality and development of TB. The improved survival is intriguing, as most of the deaths in the control group did not appear to be TB-related. This raises the possibility that the effect of isoniazid on survival may be due to more than just its effect on *M. tuberculosis*. The rates of TB in the treatment group were approximately half of those in the control group, and importantly none of the children who developed TB while on isoniazid prophylaxis had evidence of drug-resistant organisms. It will be interesting to see if these effects remain when longer term follow-up data are available. Few of the children in this study were on antiretroviral therapy, and it will be important to establish if isoniazid prophylaxis has any additional benefit to antiretroviral therapy alone as the rollout of antiretrovirals continues. Unfortunately the kind of longitudinal data that are required to fully assess the use of isoniazid for TB prophylaxis in HIV infected children cannot be provided by this study alone. Areas that still need evaluation include the effect of an intervention such as this in regions of low TB prevalence, and how the added pill burden affects compliance with antiretroviral therapy.

One could question the editorial justification for publishing a review where only one paper fulfils the criteria for inclusion, particularly when the review is conducted by the authors of that paper. However, this should not take away from the finding that on this important topic more research is urgently required. It is hoped that this review will act as a bookmark for future reviews in the expectation that further quality evidence will be obtained.

Those looking to read the principle paper from this review should note that there is an error in the citation. It is listed in the review as having been published in *The Lancet* whereas publication in fact occurred in the *British Medical Journal*.

## Declarations of Interest

None.
